# Giant Mesenteric Cavernous Lymphangioma in an Adult as a Cause of Chronic Intestinal Subocclusion: A Case Report

**DOI:** 10.7759/cureus.78934

**Published:** 2025-02-13

**Authors:** Barbara P Cab-Serrano, Victor M Ayuso-Diaz, Eduardo A Torres-Valdes, Ricardo E Chacon-Pacho, Angelica Moreno-Enriquez

**Affiliations:** 1 Surgery, Hospital Regional Elvia Carrillo Puerto, Institute for Social Security and Services for State Workers (ISSSTE), Yucatán, MEX; 2 Clinical Recruitment, Medical Care and Research, Yucatán, MEX; 3 Surgery, Elvia Carrillo Puerto Regional Hospital, Yucatán, MEX; 4 Genomic-Metabolic Unit, Marista University of Mérida, Yucatán, MEX

**Keywords:** abdominal lymphangioma, cavernous lymphangioma, chronic abdominal pain, chronic lower abdominal pain, giant abdominal tumour, intestinal subocclusion, lymphatic malformations, mesenteric lymphangioma, mesenteric tumours, postoperative complications

## Abstract

Giant mesenteric cavernous lymphangioma is a rare lymphatic malformation in adults and an uncommon cause of chronic bowel obstruction. Although these lesions are typical of the paediatric population, their occurrence in adults poses significant diagnostic challenges due to their clinical and radiological similarity to malignant neoplasms such as liposarcoma, lymphoma and gastrointestinal stromal tumours. This report describes the case of a 58-year-old man with chronic bowel obstruction in whom the initial diagnosis suggested malignancy. After surgical resection, histopathological examination confirmed the benign nature of the lesion and identified it as a mesenteric cavernous lymphangioma. The present case contributes to the understanding of this entity in adults and emphasises its importance in the differential diagnosis of chronic intestinal obstruction. It also highlights the need to integrate clinical, radiological and anatomopathological evaluation to establish an accurate diagnosis and guide appropriate therapeutic decisions, with direct implications for the management of complex abdominal pathologies.

## Introduction

Lymphatic malformations are a heterogeneous group of benign lesions that, although more common in the paediatric population, can occasionally manifest in adults [1]. Among these, mesenteric cavernous lymphangioma is one of the most unusual presentations within the abdominal cavity, characterised by dilated lymphatic cavities lined with endothelium. It is rarely diagnosed in adults, which complicates the clinical approach and differential diagnosis [2].

In the context of chronic bowel obstruction, this type of lesion may be confused with malignant neoplasms such as liposarcoma, lymphoma or gastrointestinal stromal tumour (GIST) due to its clinical features and radiological findings. These similarities may delay the accurate identification and initiation of appropriate treatment [3].

The diagnosis of mesenteric cavernous lymphangiomas requires a multidisciplinary approach including history, advanced imaging studies and ultimately histopathological confirmation. Surgical intervention is usually necessary both to confirm the diagnosis and to relieve symptoms associated with bowel compression or subocclusion [2,3]. In this report, we present the case of a 58-year-old man with chronic bowel obstruction caused by a giant mesenteric cavernous lymphangioma. This case allows us to reflect on the clinical and therapeutic characteristics of this pathology in adults, providing valuable information to optimise the diagnostic protocols and surgical management of complex abdominal conditions.

## Case presentation

We present the case of a 58-year-old male patient with a medical history of type 2 diabetes mellitus and systemic arterial hypertension, both controlled by regular medical treatment. The patient had no personal or family history of oncological disease nor a history of smoking, alcoholism or toxic substance use. He also had no history of recurrent abdominal pain or previous episodes of bowel obstruction prior to the onset of the current symptoms, which included intermittent abdominal discomfort, postprandial fullness and mild constipation over a period of approximately three months. However, there were no severe obstructive symptoms such as vomiting or significant weight loss.

The patient was admitted to the hospital due to the presence of a right flank nodule discovered during a routine physical examination. On palpation, the nodule was firm and non-tender with poorly defined margins. No clear signs of malignancy were observed, but due to the characteristics of the tumour and the possibility of underlying pathology, it was decided to perform imaging studies to obtain a more accurate diagnosis.

A contrast-enhanced thoracoabdominal-pelvic computed tomography (CT) scan was performed, showing a hypodense lesion measuring 63 × 100 × 78 mm located in the mesentery anterior to the right iliac vessels. No clear radiological signs of intestinal obstruction were identified, as there was no significant bowel dilation or air-fluid levels. However, the lesion's characteristics were suggestive of liposarcoma, leading the medical team to consider this possibility as the primary diagnosis. A guided percutaneous biopsy was planned to obtain a sample of the lesion and confirm the diagnosis.

The percutaneous biopsy was performed using an 18 G × 20 cm cutting needle, under ultrasound guidance. The puncture site was located in the right lower quadrant, avoiding major vascular structures. Only a small fragment of tissue was obtained, which, upon histopathological analysis, was insufficient for a definitive diagnosis. The sample consisted mainly of adipose tissue and chylous material. Given the absence of clear histological information, it was decided to proceed with an exploratory laparotomy to assess the lesion directly and obtain an adequate sample for a definitive diagnosis.

**Figure 1:**
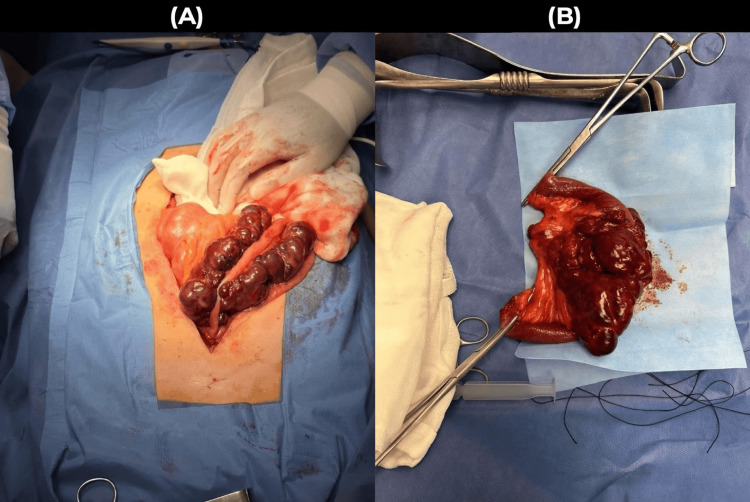
Intraoperative findings during laparotomy for the resection of a mesenteric tumour (A) Tumour location: Image showing the anatomical location of the mesenteric tumour in relation to adjacent structures prior to surgical resection. (B) Dissected mesentery and bowel with a 5 cm margin: Post-dissection view of the mesentery and bowel segment demonstrating a safe 5 cm surgical margin around the tumour site. All images were captured using a high-resolution smartphone camera to ensure detailed visualisation of the surgical findings.

At laparotomy, an approximately 20 × 15 cm tumour was observed in the small bowel (Figure 1A), located approximately 180 cm distal to the ligament of Treitz, with an additional 3 cm mesenteric lymph node located 80 cm from the ligament of Treitz. The mesentery was dissected (Figure 1B), and a segmental resection of the small bowel was performed with mechanical anastomosis using a linear stapler, ensuring a 5 cm surgical margin around the tumour site. The surgical specimen was sent for histopathological analysis.

**Figure 2:**
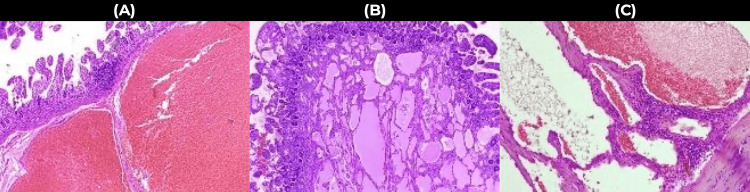
Microscopic appearance of the tumour (A) Submucosal angiolymphatic cavernous canals: Microscopic image showing the presence of dilated angiolymphatic canals within the submucosal layer, characteristic of the tumour's vascular architecture. (B) Cystic-cavernous areas: Histological section displaying cystic and cavernous spaces, indicative of the tumour's structural morphology. (C) Ischaemic necrosis: Microscopic visualisation of ischaemic necrosis within the tumour, highlighting areas of tissue degeneration and hypoxia. All images were obtained using a high-resolution microscope camera to provide detailed documentation of the tumour's histopathological features.

Histopathological analysis revealed a diagnosis of mesenteric cavernous lymphangioma measuring 13 × 10 × 8 cm, which was completely resected without evidence of malignancy. Microscopic sections showed edematous fibromuscular tissue walls with infiltration of mature lymphocytes and myofibroblasts, together with cystic-cavernous areas lined by flattened endothelial cells. The intestinal mucosa showed congestion with superficial ischaemic necrosis of the villi without significant changes at the surgical margins. Submucosal angiolymphatic cavernous channels were present, confirming a cavernous lymphangioma (Figure 2).

Postoperatively, the patient had a favourable recovery for the first 48 hours. Adequate support was provided with intravenous fluids and analgesics, and stable blood pressure levels were maintained. Initial postoperative labs showed stable haemoglobin (13.2 g/dL), leukocytes within normal limits (6,500/mm³) and normal renal function (creatinine: 0.9 mg/dL). At 24 hours postoperatively, the patient tolerated oral fluids without signs of abdominal distension or digestive complications. Bowel function was assessed by auscultation and peristalsis, which was adequate. Additionally, the patient remained afebrile with no signs of infection, and his general condition was satisfactory.

Close monitoring of haemodynamic and respiratory status was maintained, and antithrombotic prophylaxis with low-molecular-weight heparin was started according to postoperative protocols. However, on postoperative day 6, the patient developed sudden dyspnea and tachycardia. Laboratory tests showed an elevated D-dimer level (2,500 ng/ml), and arterial blood gases revealed hypoxemia (PaO₂: 58 mmHg). Given these findings, a chest CT scan was performed, confirming bilateral pulmonary thromboembolism (PTE). Additional laboratory tests, including troponin T and proBNP, were performed to assess for possible cardiac involvement, but no evidence of primary or secondary cardiac events was found. The chest CT scan further revealed that the thrombi were located in the segmental pulmonary arteries rather than the central trunk. Despite urgent administration of anticoagulants and intensive care, the patient's condition continued to deteriorate. Within 72 hours of the diagnosis of PTE, the patient suffered a cardiorespiratory arrest that could not be reversed, resulting in his death.

This case highlights the importance of a multidisciplinary diagnostic and therapeutic approach in the evaluation of rare mesenteric tumours. Despite the suspicious features of malignancy observed on imaging, definitive diagnosis was only possible through surgery and histopathological analysis. It also highlights the need for careful postoperative management, with particular attention to thromboembolic complications, which should be considered in patients with pre-existing medical and surgical conditions.

## Discussion

The distinction between vascular malformations and tumours is of fundamental importance in clinical practice, given the significant differences between them with regard to diagnosis and management. Notwithstanding these differences, there is a tendency to conflate the two, which can result in inappropriate or delayed management. Vascular tumours are defined as acquired proliferations of newly formed blood vessels, whereas vascular malformations are congenital anomalies characterised by non-proliferative vessels of dysmorphic morphology. Despite the common features shared by these two entities, the distinction between them is paramount for effective diagnostic and therapeutic approaches [4].

Among the vascular malformations, lymphangiomas are benign formations of the lymphatic vessels that occur in two main forms: simple lymphangiomas and cavernous lymphangiomas. Simple lymphangiomas, or capillary lymphangiomas, are small, slightly elevated or pedunculated lesions, 1-2 cm in size, commonly found on the head, neck and axilla. Histologically, these lesions consist of a network of endothelium-limited spaces and are distinguished from capillary channels by the absence of blood vessels [5]. In contrast, cavernous lymphangiomas, also known as cystic hygromas, are much larger lesions composed of dilated lymphatic spaces separated by a connective tissue stroma with lymphoid aggregates. These lesions are most commonly found in the neck and axilla of paediatric patients, although they are also rarely found in the retroperitoneum. Due to their size and location, cavernous lymphangiomas can cause significant deformity and surgical complications because their margins are not well defined, making complete resection difficult [5,6].

The occurrence of lymphatic malformations in the abdominal region is uncommon, constituting a mere 5% of all documented cases. The preponderance of cases involving the mesenteric location is particularly noteworthy. A higher incidence of abdominal lymphangiomas has been observed in males, exhibiting a ratio of 3:2 [7,8]. In the paediatric population, mesenteric lymphangiomas may account for up to 45% of abdominal cases, with involvement of the small bowel mesentery being the most common, followed by the omentum, mesocolon and retroperitoneum [9]. Symptomatology in these cases can be asymptomatic in up to 50% of cases, and diagnosis is usually made incidentally through imaging studies or when the patient presents with recurrent abdominal pain. However, the most common presentation is a mechanical obstructive syndrome caused by compression, angulation or volvulus of the bowel adjacent to the tumour. In some cases, bleeding due to erosion of the cystic tumour walls has been reported [10,11].

A study of 145 cases of paediatric mesenteric lymphangiomas revealed that these tumours can manifest as large cysts in the mesentery, characterised by a cavernous component that disrupts the muscular and submucosal layers of the bowel, resulting in haemorrhage, necrosis and acute inflammatory changes in the mesentery. This dissemination and separation of mesenteric structures have also been observed in other sites and have been shown to become more severe with increasing tumour size, thereby increasing the complexity of the condition and the severity of the consequences for the patient [12]. Furthermore, in studies carried out in paediatric hospitals, mesenteric lymphangiomas have been diagnosed in patients aged between one month and seven years, usually with symptoms of intestinal obstruction, whereas abdominal lymphangiomas are less common in patients over 14 years of age [13].

The aetiology of lymphangiomas appears to be a primary malformation during embryological development in which the lymphatic tissue is sequestered, thus explaining the preponderance of lymphatic malformations in children. However, it has been hypothesised that factors such as abdominal trauma, lymphatic obstruction, inflammatory processes, surgery or radiotherapy may contribute to the secondary development of these tumours. This could account for the occurrence of lymphatic malformations in patients over 60 years of age who previously had normal imaging studies [14,15].

The diagnosis of mesenteric lymphangiomas is primarily determined by imaging techniques, with CT demonstrating unilocular or multilocular masses with septa of variable thickness. The utilisation of contrast medium enhances the visualisation of the tumour walls, facilitating the assessment of its location, extent and relationship to neighbouring organs. In certain instances, angiography may reveal the displacement or reduction of the arterial lumen by the tumour; however, it does not permit direct visualisation of the lymphangioma's vasculature. Furthermore, preoperative fine needle aspiration can confirm the diagnosis by obtaining a milky fluid containing lymphoid cells, a useful procedure when technically feasible [16].

The treatment of mesenteric lymphangiomas is predominantly surgical in nature, with the primary objective being the complete resection of the tumour. However, this objective may be hindered by the unencapsulated nature and indistinct margins characteristic of cavernous lymphangiomas. The rationale for maintaining a surgical margin of 5 cm in such cases stems from the risk of incomplete resection, a consequence of these tumours' propensity to infiltrate surrounding structures, thereby complicating the clear definition of their boundaries. The 5 cm margin strategy is also adopted in similar surgical scenarios to ensure negative margins and reduce the likelihood of local recurrence. In addition, although lymphoscintigraphy has been proposed as a non-invasive method to evaluate lymphatic flow and detect lymphatic malformations, its role in the diagnosis of mesenteric lymphangiomas remains limited due to variable sensitivity and the lack of standardised protocols, which makes CT and magnetic resonance imaging (MRI) the preferred imaging modalities. In the context of core biopsy, while its efficacy in confirming the diagnosis of lymphangiomas is acknowledged, its role is not without limitations. In particular, its application is often precluded in cases involving large tumours or those exhibiting complex structural patterns. In the present case, the core biopsy yielded inconclusive results, a consequence of the tumour's low cellularity and heterogeneous composition. Furthermore, the potential risks of tumour seeding and sampling error should be considered, especially when the lesion is difficult to access or effectively aspirate. While core biopsy remains a useful diagnostic tool in selected scenarios, its limitations in large mesenteric lymphangiomas often necessitate direct surgical exploration to obtain definitive histopathological confirmation [17,18].

This case underscores the importance of a multidisciplinary diagnostic and therapeutic approach in the evaluation of rare mesenteric tumours. Despite the suspicious features of malignancy observed on imaging, a definitive diagnosis was only possible through surgical exploration and histopathological analysis. Furthermore, the case emphasises the necessity for meticulous postoperative management, with particular emphasis on the potential for thromboembolic complications, which should be considered in patients with pre-existing medical and surgical conditions.

## Conclusions

Mesenteric cavernous lymphangioma is a rare pathology, especially in adults, and its aetiology is considered to be mainly idiopathic, although factors such as minor trauma could have influenced the formation of the lymphoid conglomerate. Diagnosis by CT scan is consistent with that described in the literature, while needle biopsy, although less common, was not successful in this case. Surgical treatment by complete resection of the lesion remains the standard therapeutic approach, although cases documented in the literature do not present a similarly precise history. This case highlights the importance of considering mesenteric cavernous lymphangioma in the differential diagnosis of patients with chronic abdominal pain or recurrent episodes of subocclusion, regardless of age, to improve diagnostic accuracy and avoid delays in definitive surgical treatment.
